# Novel approach for quantification of multiple immunofluorescent signals using histograms and 2D plot profiling of whole-section panoramic images

**DOI:** 10.1038/s41598-021-88101-1

**Published:** 2021-04-21

**Authors:** Roko Duplancic, Darko Kero

**Affiliations:** 1 Study Program of Dental Medicine, School of Medicine, University of Split, Soltanska 2, 21000 Split, Croatia; 2 Department of Anatomy, Histology and Embryology, Laboratory for Early Human Development, School of Medicine, University of Split, Soltanska 2, 21000 Split, Croatia

**Keywords:** Computational biology and bioinformatics, Imaging, Immunological techniques, Immunohistochemistry, Immunology, Chronic inflammation, Biomarkers, Diagnostic markers, Pathogenesis, Chronic inflammation

## Abstract

We describe a novel approach for quantification and colocalization of immunofluorescence (IF) signals of multiple markers on high-resolution panoramic images of serial histological sections utilizing standard staining techniques and readily available software for image processing and analysis. Human gingiva samples stained with primary antibodies against the common leukocyte antigen CD45 and factors related to heparan sulfate glycosaminoglycans (HS GAG) were used. Expression domains and spatial gradients of IF signals were quantified by histograms and 2D plot profiles, respectively. The importance of histomorphometric profiling of tissue samples and IF signal thresholding is elaborated. This approach to quantification of IF staining utilizes pixel (px) counts and comparison of px grey value (GV) or luminance. No cell counting is applied either to determine the cellular content of a given histological section nor the number of cells positive to the primary antibody of interest. There is no selection of multiple Regions-Of-Interest (ROIs) since the entire histological section is quantified. Although the standard IF staining protocol is applied, the data output enables colocalization of multiple markers (up to 30) from a given histological sample. This can serve as an alternative for colocalization of IF staining of multiple primary antibodies based on repeating cycles of staining of the same histological section since those techniques require non standard staining protocols and sophisticated equipment that can be out of reach for small laboratories in academic settings. Combined with the data from ontological bases, this approach to quantification of IF enables creation of in silico virtual disease models.

## Introduction

The immunofluorescence (IF) (and immunohistochemistry (IHC) in general) has long been recognized as one of the fundamental methods for biomedical research. Following the advent of antibodies targeted against specific proteins (and to a certain degree other classes of molecules such as glycans), IF is being regularly applied as a complementary method for the molecular profiling of tissue samples, which in turn is useful for the diagnostic purposes (subtyping of diseases such as tumors, inflammatory diseases, autoimmune disorders), and for the evaluation of outcomes of different experimental procedures performed in basic biomedical research. However, the ever increasing knowledge about the complexity of molecular regulatory networks derived from numerous experiments on knockout animals, functional and high throughput studies based on tissue homogenates (genome and proteome sequencing) puts additional demands on IF/IHC-based research of human tissue samples^1^. To understand how a certain protein might be relevant to any given cellular or tissue process, it is not sufficient to simply observe if the signal (or staining) of that protein is present or absent in the sample of interest, but must also be disclosed in quantifiable parameters 'How much?' and 'Where in the cell/tissue?' the staining is present. While the molecular function of a protein is determined by its biochemical properties, the protein's cellular/tissue function is influenced by its spatial relation to other proteins present in the same cellular/tissue compartment, which comprise either a well-defined functional group and/or participate in the inter-connected regulatory pathways^2^.

IF signal have three main properties. The first one is the expression pattern which can be nuclear or non-nuclear (cytoplasmic, cell surface). The second one is its expression domain, i.e. the area occupied by IF signal. The third property of IF signal relates to its spatial gradient, i.e. how the IF signal is distributed within the cell/tissue based on the variations of its overall intensity. At present, there are software tools for quantification of all three of these properties, but while the conventional computer-assisted scoring systems for quantification of staining are able to turn the expression patterns and expression domains into quantifiable parameters, they still fall short on quantification of IF signals' spatial gradients^3^. This does not pose much of a problem for quantification of protein-markers expressed in the well-defined cell compartments (cell nuclei), or when their expression is localized to specific tissue structures and thus able to be analyzed within smaller Regions-Of-Interest (ROIs) determined by investigator^4^. However, the quantification of IF signals from ubiquitously expressed markers with non-nuclear expression patterns requires different approach. This is not only due to their generally large expression domains (which in conventional approach would necessitate the selection of increasing number of ROIs), but also because their spatial gradients are extremely important indicator of biological function^5,6^.

Here we describe a novel approach for quantification of expression domains and spatial gradients of multiple IF signals. To demonstrate the mechanics of the approach, we used human gingiva samples stained with primary antibodies against cell surface heparan sulfate proteoglycan (HSPG) syndecan 1 (Sdc1), heparan sulfate glycosaminoglycan (HS GAG), HS GAG-biosynthesis proteins and common leukocyte antigen CD45 (inflammatory cell marker). IF signals were quantified on the high-resolution whole-section panoramic images. In this approach, we utilize the readily available software for digital image editing and digital image analysis. This approach to quantification of IF staining utilizes pixel (px) counts and comparison of px grey value (GV) or luminance for the analysis of afforementioned properties of IF staining. No cell counting is applied either to determine the cellular content of a given histological section nor the number of cells positive to the primary antibody of interest. There is also no need for selection of multiple ROIs since the entire area of histological section is quantified. Although the standard IF staining protocol is applied, the data output enables colocalization of multiple markers (up to 30) from a given histological sample. This can serve as an alternative for colocalization of IF staining of multiple markers based on repeating cycles of staining of the same histological section since those techniques require non standard staining protocols and sophisticated equipment that can be out of reach for small laboratories in academic settings.

## Results

### Histomorphometry—whole-section area, fraction areas, cellularity

**Figure 1:**
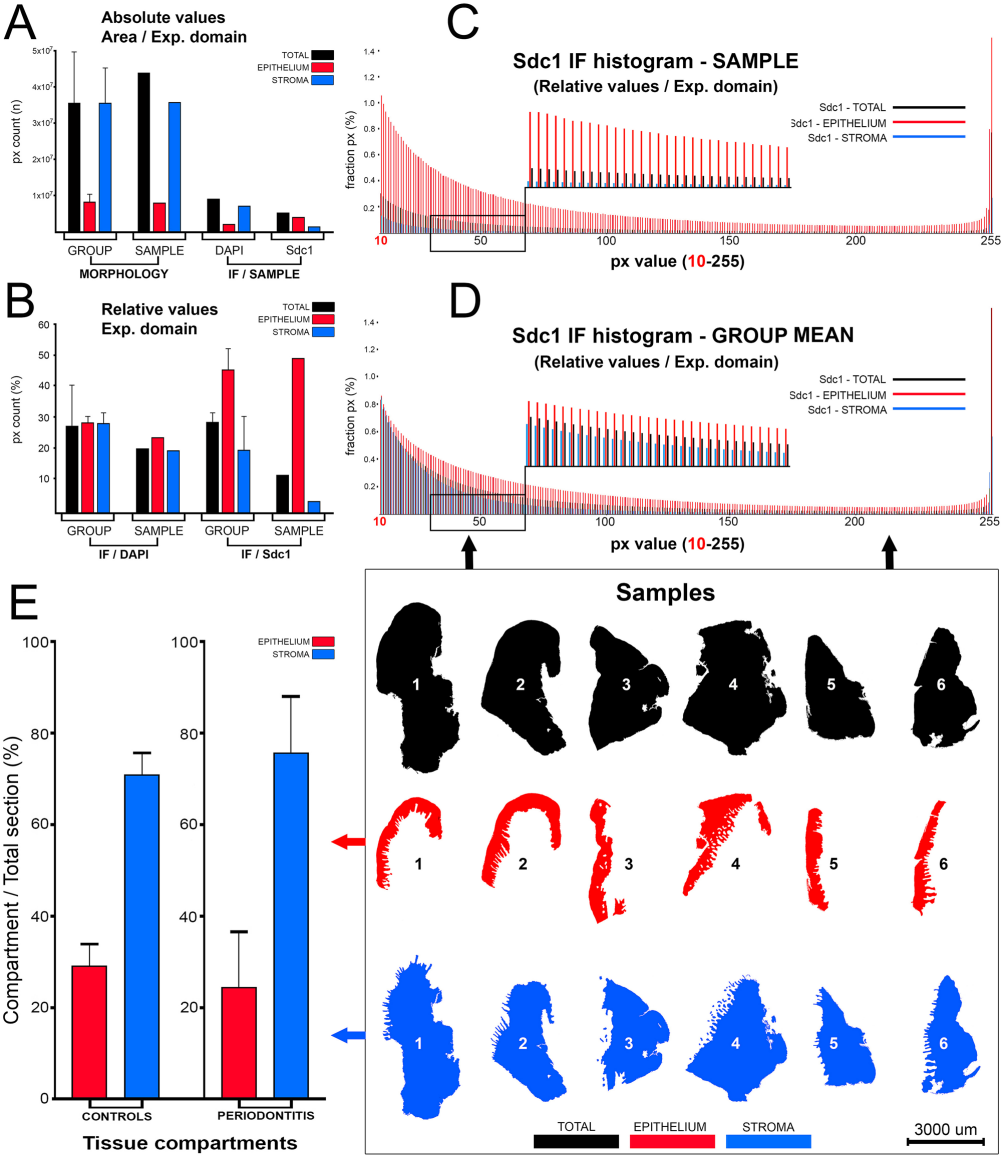
Formatting of histogram data from panoramic IF images of Sdc1 and DAPI IF signals (sample: DK-JN19-CHP; whole-section area—12.89 mm^2^; epithelial fraction area—18.42%; stromal fraction area—81.58%) for statistical analysis of histomorphometry and expression domains; (*Scale bar:* 3000 µm). The numerical values of expression domains are presented cumulatively as absolute (**A**) and relative px counts (**B**,**E**), or fractionally (**C**,**D**) where the proportion of pxs is calculated for each px GV on 10–255 scale (x-axis) either for the whole section or particular tissue compartment. The advantage of this approach is that not only the total numerical value of expression domains can be calculated, but also the intensity of expression with regard to variations in px GV. Since the histogram output is congruent (total of 246 values), histograms with relative values can be used for calculation of investigated factors' expression domains as group means (**D**, thick black arrows) and compared between different groups of samples. In contrast to absolute values, the use of relative values (proportions/percentages) is necassary due to variations in size between sections from different samples (in frame 1–6). Accordingly, certain histomorphometric parameters might also be of importance in subsequent statistical analysis of IF signals' expression domains—these parameters are related to cellularity (presented here as cumulative values of DAPI expression domains (**A**,**B**)) and the overall tissue structure (epithelial and stromal compartment area fractions). On a between-group level, gingival samples from control and test (periodontitis) group have similar structure (**E**) with regard to the fraction areas of epithelial (29.13% vs. 24.27%) and stromal tissue compartments (70.91% vs. 75.72%) (*t*.-test: *P* = 0.3075; *P* > 0.1 (α = 0.1)). Thus, the statistical comparison of mean of IF signals' expression domains between the two groups is not confounded by the potential over-estimation of the expression domains of factors with predominant expression in particular tissue compartment. (Image created in Adobe Photoshop CC 2014, ver. 6.3; https://www.adobe.com/products/photoshop.html).

**Figure 2:**
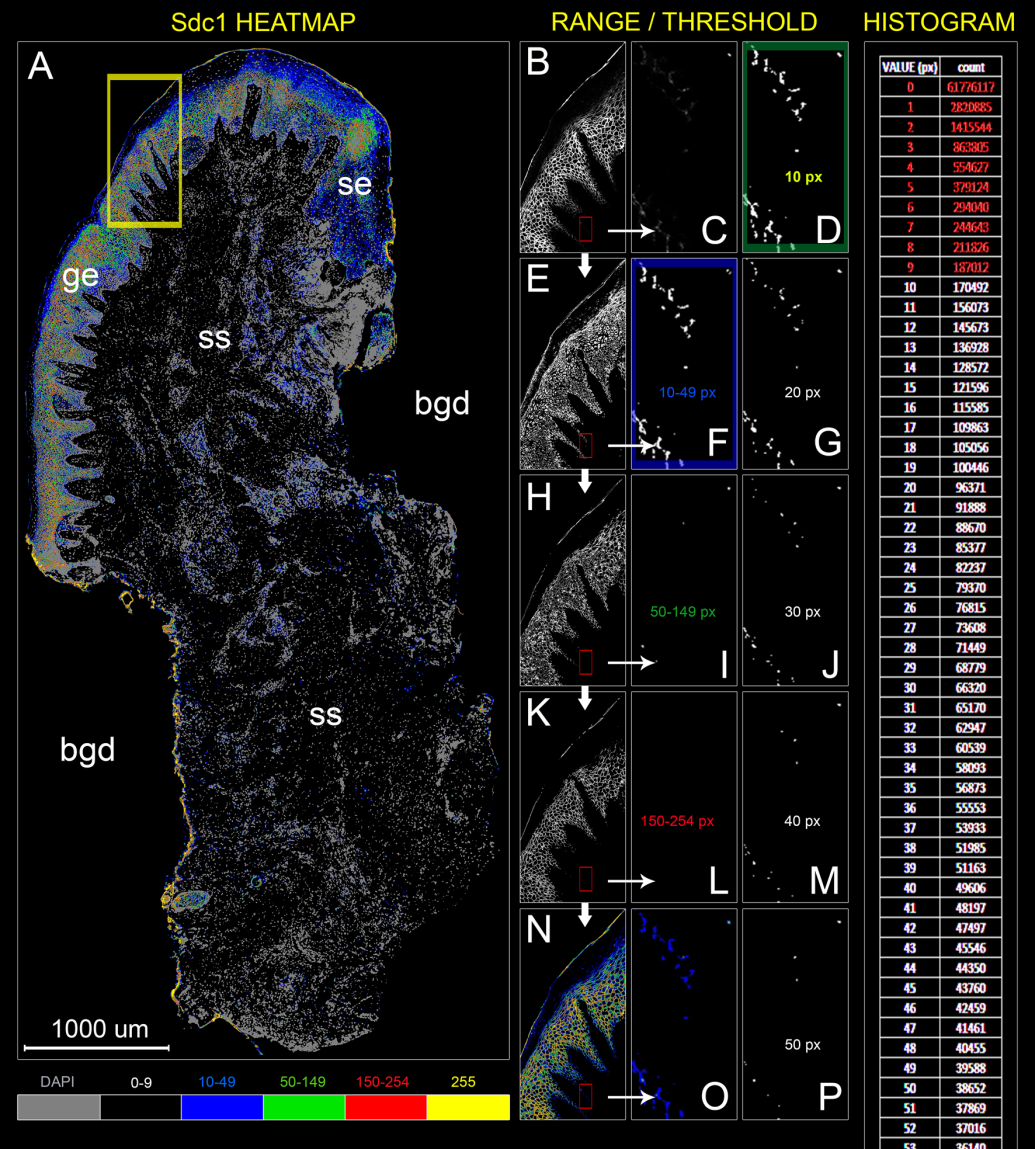
Panoramic four-color heatmap for visualization of IF signal dynamic range (**A**) and px GV threshold setting (**B**–**P**) of IF signals from anti-Sdc1 primary antibody staining of gingiva from patient with advanced generalized periodontitis (sample: DK-JN19-CHP). Designations: *bgd* background, *ge* gingival epithelium, *se* gingival sulcus epithelium, *ss* subepithelial stroma. (Heatmap intensity range: black (0–9 px), blue (10–49 px), green (50–149 px), red (150–254 px) and yellow (255 px); grey—DAPI); (*Magnification:* × 10 (**A**–**P**); *scale bar:* 1000 µm). To determine the minimal value of IF signal dynamic range (px GV threshold), a series of 8-bit binary threshold images from the original panoramic IF image is made (starting from 1 px GV and higher at 1 px increments). The first 8-bit threshold image with background at 0 px GV is selected. In this case this is at px GV of 10. Subsequently, another series of 8-bit threshold images is made (threshold range) (thick white arrows; **E**,**H**,**K**) and matched (thin arrows) with regions on the original IF image (**B**, corresponding to yellow frame) containing strong (**N**) and weak IF signals (small red frames; **C**,**O**). Once the 8-bit threshold range image (**F**,**I**,**L**) which best fits the spatial distribution of weak signals is determined (**F**, blue frame), the third series of 8-bit threshold images is made (**D**,**G**,**J**,**M**,**P**). Spatial correlation between the several within-range px GV threshold areas and weak IF signals is then determined by linear regression using 2D plot profile values (THRLD_10_ *R*^*2*^ = 0.79, *P* = 4.64 × 10^–61^; THRLD_20_ *R*^*2*^ = 0.68, *P* = 3.49 × 10^–45^; THRLD_30_ *R*^*2*^ = 0.46, *P* = 8.77 × 10^–26^; THRLD_40_ *R*^*2*^ = 0.41, *P* = 9.3 × 10^–22^; THRLD_50_ *R*^*2*^ = 0.3, *P* = 1.45 × 10^–15^). The 8-bit threshold image with the highest coefficient of determination (*R*^*2*^) (**D**, green frame) is used to set the IF signal threshold, in this case at px GV of 10. This is a cut-off for panoramic IF image histograms (far right). Counts of px corresponding to each GV below the threshold are excluded from the calculation. (Image created in Adobe Photoshop CC 2014, ver. 6.3; https://www.adobe.com/products/photoshop.html).

The analysis of expression domains of IF signals betwen groups of samples based on panoramic images with no selection of particular ROIs can be confounded by differences in histomorphometric profiles of the samples (Fig. 1A,B,E). The first counfounding factor is size—histological sections vary in size (even if they come from the same sample). Thus, instead of quantifying parameters in absolute values, relative values such as percentages or proportions should be used (Fig. 1A,B). The second confounding factor is related to the basic histological structure of the investigated tissue, which in this case is gingiva. Gingiva is comprised of two tissue compartments—epithelial compartment (gingival epithelium, sulcus epithelium and, in diseased samples, pocket epithelium) which encloses the connective tissue of stromal compartment. The substantial difference of group mean values of fraction areas of tissue compartments (or their ratios) presents a significant bias for statistical analysis of expression domains of IF signals between groups of samples if they, for instance, belong to a marker whose expression is more restricted to a particular tissue compartment—a good example is Sdc1, which is mainly expressed by epithelial cells and usually referred to as the epithelial HSPG (Heparan Sulfate ProteoGlycan)^7^. Basic histomorphometry was performed on both panoramic H/E images and panoramic IF images of histological sections from 40 samples of human gingiva (20 from healthy donors and 20 from patients with advanced generalized periodontitis). The following parameters were measured and analyzed by descriptive statistics and *t*-test: whole-section areas, fraction areas of tissue compartments (epithelium/stroma). The mean whole-section area of control gingiva samples was 9.29 mm^2^ (SD = ±4.63 mm^2^) compared with 12.21 mm^2^ (SD = ±3.21 mm^2^) mean whole-section area of diseased gingiva samples. Fraction areas and group means of fraction areas were expressed as relative values in percentages. The mean epithelial and stromal fraction areas of control gingiva samples were 29.13% (SD = ±12.95%) (epithelium) and 70.91% (SD = ±14.91%) (stroma), whereas in diseased gingiva samples were 24.27% (SD = ±12.34%) (epithelium) and 75.72% (SD = 12.34%) (stroma). There were no statistically significant difference of mean epithelial and stromal fraction areas between groups (*t*-test: *P* = 0.3075; *P* > 0.1 (α = 0.1)). The measurement of cellularity in each sample was based on adding up relative values of fractional px counts sorted on a 10-255 px grey value scale from histograms of whole section and tissue compartment panoramic IF images of DAPI staining (Figs. 1, 2A,B). Thus, the expression domain of DAPI can be used to present the cellular content of histological sections—as a proportion of the whole-section or tissue compartment area covered by cell nuclei. The mean group value of the whole-section cellularity of control and diseased gingiva samples was 26.08% (SD = ±10.07%) and 30.47% (SD = ±7.51%), respectively. No statistically significant difference in tissue cellularity was found between groups (*t*-test: *P* = 0.1989; *P* > 0.01 (α = 0.01)). Also, no statistically significant difference between groups was found when fractional cellularity of epithelial tissue compartments was compared (12.53% vs. 10.51% in epithelium; *t*-test: *P* = 1.833; *P* > 0.01 (α = 0.01)). However, the fractional cellularity of stromal compartments was significantly different (13.55% vs. 19.95%; *t*-test: *P* = 0.002; *P* < 0.01 (α = 0.01)).

### Quantification of expression domains of IF signals

Mean group values of expression domains of IF signals for whole-section, epithelial and stromal tissue compartments were analysed from histograms of panoramic IF images. The comparison of expression domain of Sdc1 between healthy and diseased gingiva samples is provided as an example. In order to compensate for differences in size between different samples, fractional px counts were recalulated as percentages from the whole-section area and tissue compartment areas (epithelial, stromal) (Fig. 1C–E). The mean whole-section Sdc1 expression domain in healthy gingiva was 20.66% (SD = ±3.08%) and in diseased gingiva 29.04% (SD ±4.97%). The mean epithelial expression domains were 46.64% (SD = ±2.76%) in healthy gingiva and 46.14% (SD = ±3.01%) in diseased gingiva, whereas the mean stromal expression domains were 7.85% (SD = ±2.54%) in healthy gingiva and 20.13% (SD = ±3.11%) in diseased gingiva. When compared between groups of samples, no statistically significant difference was found for the whole-section (ANOVA: *P* = 0.08751; *P* > 0.01 (α = 0.01)) and epithelial (ANOVA: *P* = 0.96283; *P* > 0.01 (α = 0.01)) expression domains, however there was statistically significant difference between the stromal expression domains (ANOVA: *P* = 1.1×10^−6^; *P* < 0.01 (α = 0.01)). The mean expression domain of inflammatory cell marker CD45 in diseased gingiva was found to be significantly increased compared with healthy gingiva (fourfold increase) (ANOVA: *P* = 2.7×10^−8^; *P* < 0.01 (α = 0.01)).

When using ANOVA to compare the expression of investigated marker between samples or group of samples represented as histograms of IF staining, two parameters need to deviate in order to detect statistically significant difference—firstly, the total value of px counts (either absolute or relative) from histograms need to be different (corresponding to differences in size of expression domain); and secondly, the distribution of px counts on a 10–255 px GV scale (corresponding to the overall intensity of staining) should display difference in variance. Since the information of the actual size of expression domain and the overall intensity of staining is included in histograms, there is no need for tranformation of data into expression indices. Furthermore, the number of data points from histograms (total of 246 with the px GV threshold set at 10) is sufficient to enable the use of parametric tests for statistical analysis with higher level of confidence.

### Quantification and colocalization of spatial gradients of multiple IF signals

**Figure 3:**
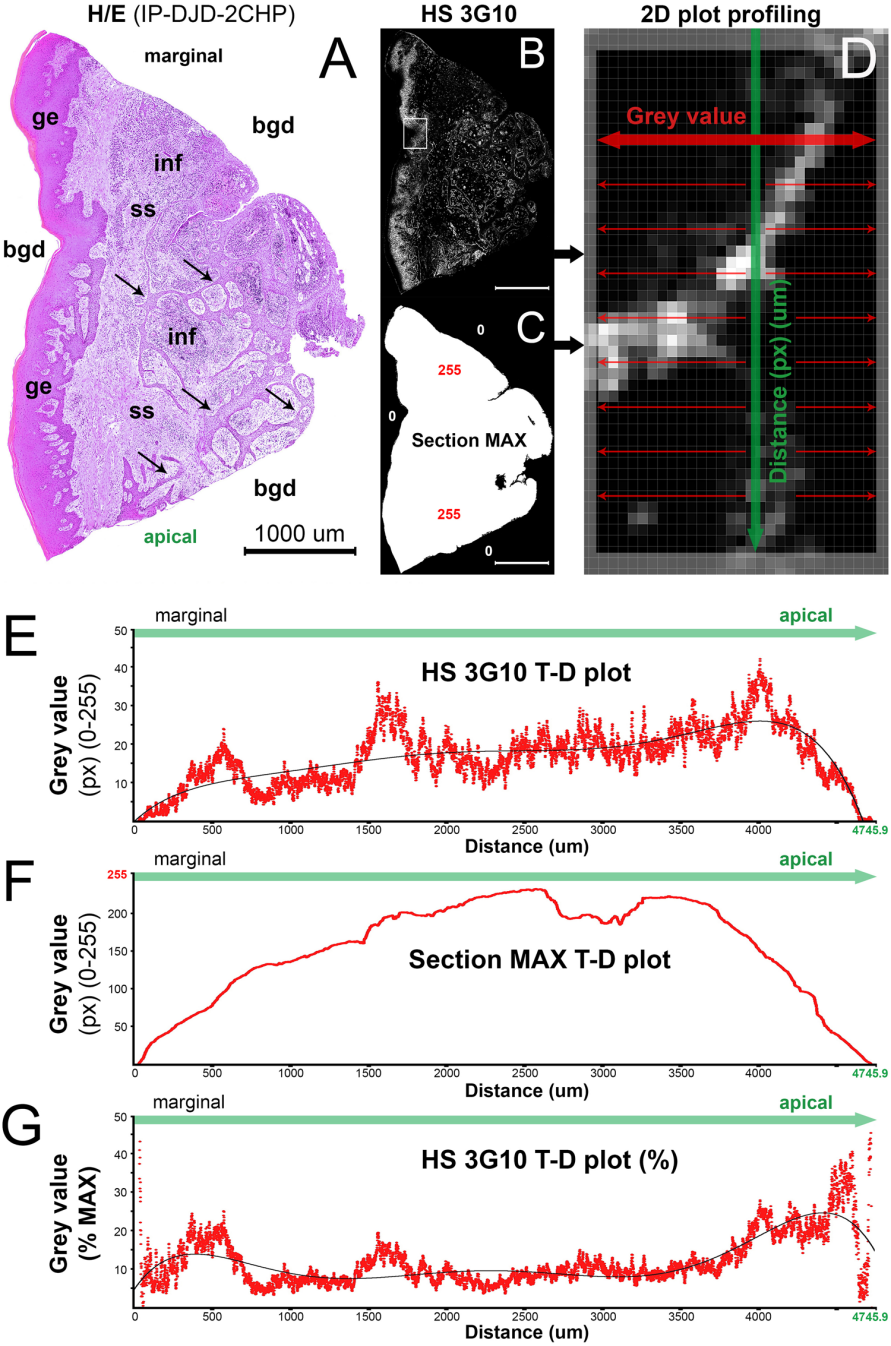
2D plot profiling of spatial gradient of anti-HS 3G10 IF signals (**B**–**F**) in panoramic IF image (dimensions: 5949 × 8800 px) of histological section of gingiva from patient with advanced generalized periodontitis (**A**) (sample: IP-DJD-2CHP; whole section area—9.38 mm^2^; epithelial fraction area—37.79%; stromal fraction area—62.21%). Designations: *bgd* background, *ge* gingival epithelium, *ss* subepithelial stroma, *inf* inflammatory infiltrate; thin arrows point to pseudo-epithelial hyperplasia with rete pegs extended throughout the subepithelial stroma as a feature of active inflammation. (*Magnification*: × 10 (**B**,**C**) and × 20 (**A**); *scale bar*: 1000 µm). To quantify the spatial gradient of a particular IF signal, the panoramic IF image (**B**) and corresponding whole-section panoramic image (C) need to be profiled (thick black arrows) using ImageJ 2D plot profiler option. 2D plot profiler calculates the mean px GV (on a 0–255 scale) for each px-wide row (two-way red arrows) (D, magnified detail from the framed region on B with px grid), from top to the bottom of the panoramic IF image (T-D plot; green arrow) which corresponds to scanning direction (from marginal toward apical portion of gingiva sample). The mean px GV for each row is calculated from a number of GVs of individual pxs (corresponding to the panoramic IF image width) producing the number of mean px GV data points (corresponding to the panoramic image height) plotted on T-D plots (E, F). The x-axis is calibrated to fit the µm scale – at 10 × magnification (scan resolution is 0.53937 µm/px; total scanning distance of 4745.9 µm). To exclude the background pxs with 0 GV from the calculation, the IF signal mean px GVs (E) need to be recalculated as relative px GVs expressed in proportions/percentages from the maximal mean px GV (F, G). The binary whole-section area image is used as a reference since it displays pxs with two GVs – 0 (background) and 255 (IF signals within the histological section area (C). Thus, the influence of all pxs locateted outside of the histological section on the T-D plot profile output is removed. (Image created in Adobe Photoshop CC 2014, ver. 6.3; https://www.adobe.com/products/photoshop.html).

**Figure 4:**
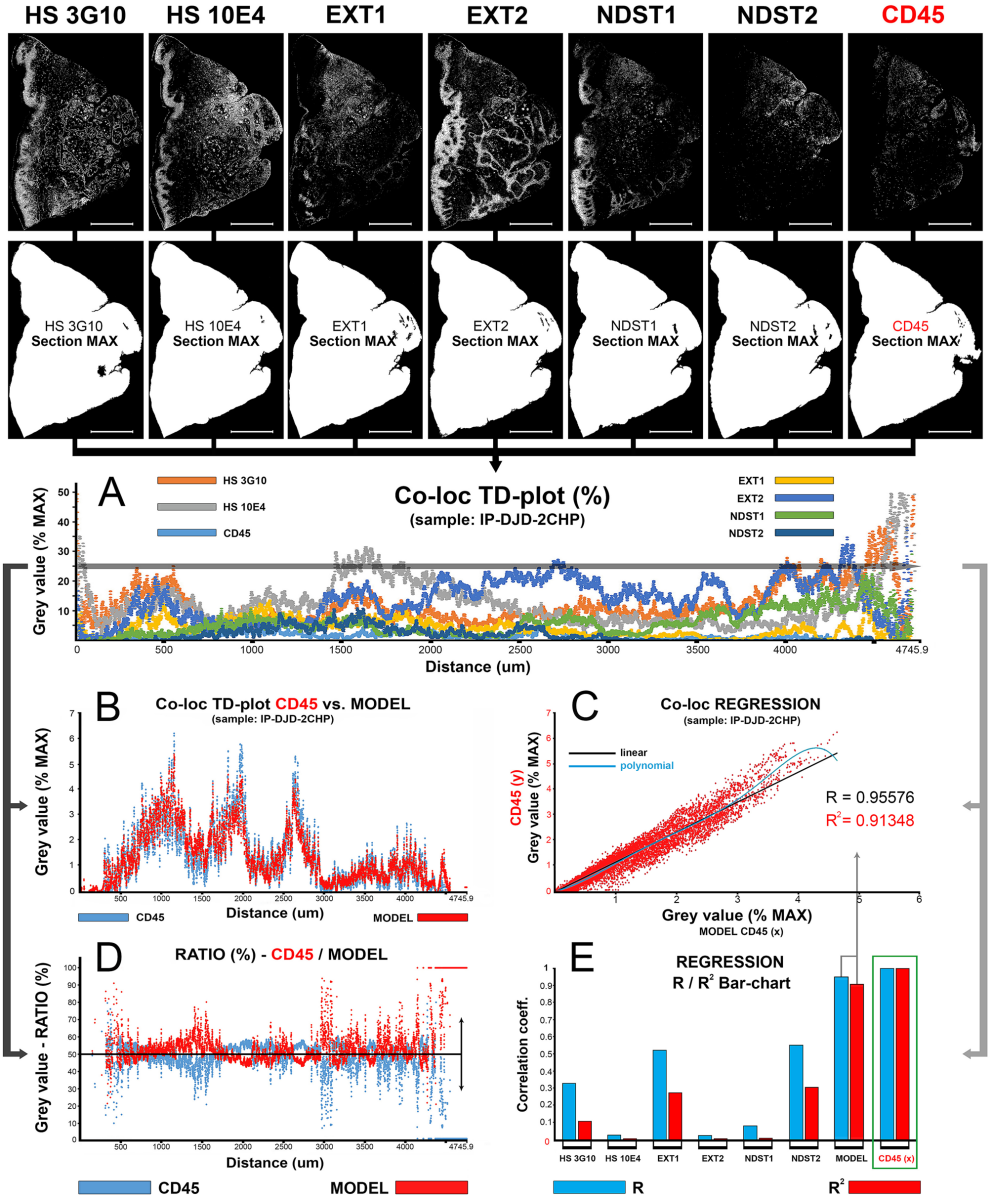
2D plot profiling for quantification and correlation of spatial gradients of IF signals from primary antibodies against HS GAG, HS GAG biosynthesis enzymes and common leukocyte antigen (CD45) in histological sections of gingiva from patient with advanced generalized periodontitis (sample: IP-DJD-2CHP). (*Magnification*: × 10; *scale bar*: 1000 µm). Spatial gradients of IF signals are plotted simultaneously on a single T-D plot (**A**) to devise the regression model. The purpose of the model is to disclose to how the presence of inflammatory cell infiltrate correlates with the expression of HS GAG and HS GAG biosynthesis enzymes. The spatial gradient for CD45 IF signal is set as dependent variable (y), whereas the spatial gradients for IF signals from HS 3G10, HS 10E4, EXT1, EXT2, NDST1 and NDST1 are designated as independent variables (x). Based on the regression function, a predicted CD45 spatial gradient is calculated (MODEL CD45) and plotted with the actual values of CD45 spatial gradient on several different plots (**B**–**D**) to check for the goodness of fit. The model is statistically significant (α = 1 × 10^–8^, *P* < 1 × 10^–8^) and reveals that the presence of inflammatory infiltrate can be well correlated with the expression of HS GAG and HS GAG biosynthesis enzymes (linear model: R = 0.95576; R^2^ = 0.91348; polynomial curve fitting: R = 0.96883; R^2^ = 0.93844). As visualized on the bar-chart of correlation coefficients (**E**), the deviation of the model (**C**,**D**) can be attributed to the low individual correlation of particular independent variables such as HS 3G10 (R = 0.32808; R^2^ = 0.10763), HS 10E4 (R = 0.02445; R^2^ = 0.00059), EXT2 (R = 0.02191; R^2^ = 0.00048) and NDST1 (R = 0.07752; R^2^ = 0.00601). IF signals of HS 3G10, HS 10E4, EXT2 and NSDT1 have larger expression domains and display different patterns than those of EXT1 and NDST2 (more confined to stromal compartment with CD45) (top row). Thus, the spatial gradients of EXT1, NDST2 display stronger correlation (EXT1–R = 0.52064; R^2^ = 0.27107) (NDST2–R = 0.55022; R^2^ = 0.30275) with CD45. (Image created in Adobe Photoshop CC 2014, ver. 6.3; https://www.adobe.com/products/photoshop.html).

2D plot profiling was used for quantification of spatial gradients of IF signals (Fig. 3). In this case, spatial gradients of IF signals from seven different factors (HS 3G10, HS 10E4, EXT1, EXT2, NDST1, NDST2 and CD45) detected by staining of a single sample of diseased gingiva were co-localized by plotting data from their individual T-D (top-down) 2D plots on the same graph (Fig. 4). Six simple linear regression models (each containing 8800 data points from T-D plots) were created with CD45 as dependent variable (y-axis), and other factors as independent variables (x-axis). The purpose of these models was to disclose the spatial overlap of the expression of investigated factors with the presence of stromal inflammatory infiltrate (CD45 IF signals). The correlation (R) and determination coefficients (R^2^) vary depending on the amount of spatial overlap or colocalization of IF signals. Since most of the investigated factors are ubiquitously expressed in gingiva and not only in the areas with inflammatory infiltrate, R and R^2^ values were closer to zero than 1 being the lowest for EXT2 (R = 0.0219; R^2^ = 0.0004; *P* < 1×10^−8^ (α = 1×10^−8^)) and highest for NDST2 (R = 0.5502; R^2^ = 0.3027; *P* < 1×10^−8^ (α = 1×10^−8^)) (Fig. 4D). Ultimately, a multiple linear regression model with CD45 as dependent variable and investigated factors as six independent variables was created. This was done in order to predict how the investigated factors affect the presence of inflammatory infiltrate—HS GAGs and related enzymes are known regulators of many cellular processes including the inflammatory response in which they can have both pro- and anti-inflammatory roles^8–13^. The model shows high correlation and statistical significance (R = 0.96883; R^2^ = 0.93844; *P* < 1×10^−8^ (α = 1×10^−8^)) (Fig. 4B–D). For calculation of the effect, a consecutive virtual knockout (VKO) was performed. In VKO, all values in T–D plot profiles of indepndent variable of interest are replaced with zeros having the mean px GV from the T–D profile of dependent variable re-calculated and compared with baseline value. According to VKO, some factors were predicted to exert pro-inflammatory effect (most pronounced being that of EXT1 with 2,5-fold decrease in mean CD45 grey value after virtual knockout), whereas others were anti-inflammatory (e.g. 1,5-fold increase in total CD45 grey value after virtual knockout of EXT2). The predictions from this model are far from being conclusive because the model is based on data from a single sample. However, it serves well to demonstrate how 2D plot profiling can be used in creation of models for prediction of behavior of biological systems. The underlying assumption is that the biological functions of molecular components of tissue are closely related to their spatial distribution.

The precision of colocalization of multiple factors and statistical models based on 2D plot profiling can be confounded by the differences in shape and cellular content of serial sections within sample. Thus, the number of sections compatible for multiple colocalization analysis was assessed by correlating spatial gradients of DAPI staining (Table 1). DAPI staining is visible in all serial sections because it is routinely performed as the background IF staining. Ideally, the spatial gradients of particular staining performed consecutively on serial sections from the same sample should display perfect linear correlation if the sections have identical shape and cell content. According to the analysis of DAPI staining, near perfect correlation can be observed for up to 30 serial sections from individual gingiva samples (R = 0.9996; R^2^ = 0.9993; *P* ~ 0). This also means that if each of those serial sections is successfully stained with different primary antibody, spatial gradients of up to 30 different factors could be colocalized by 2D plot profiling without the introduction of significant sampling error.

**Table 1:** Compatibility of serial sections for in silico colocalization and regression analysis of multiple IF signals based on correlation of spatial gradients of DAPI staining in whole-section panoramic images (sample: IP-DJD-2CHP).

Simple linear regression – DAPI staining T–D plots							
Sections	R^2^	Std. Error†	Coefficient	CI‡		Significance^§^	
Sequence*				Lower	Upper	F value	*P*
REF (x)/(y)	1	1.9553 × 10^–14^	1	1	1	2.6303 × 10^34^	0
5th (x)	0.9989	1.0879	1.0022	1.0007	1.0038	8.4859 × 10^6^	0
10th (x)	0.9980	1.4998	1.0082	1.0062	1.0103	4.4611 × 10^6^	0
15th (x)	0.9943	2.5441	0.9883	0.9848	0.9919	1.5447 × 10^6^	0
20th (x)	0.9940	2.6178	1.0091	1.0053	1.0127	1.4584 × 10^6^	0
25th (x)	0.9846	4.1924	1.0086	1.0027	1.0146	5.6328 × 10^5^	0
30th (x)	0.98141	4.6084	1.0317	1.0251	1.0384	4.6466 × 10^5^	0

*Serial number of histological section stained with DAPI—the first section was used as reference (REF) (dependent variable—y) for auto-correlation (reference model); DAPI staining of every fifth consecutive section up to 30^th^ section (independent variables—x) was correlated with the DAPI staining of the referent section.

^†^Standard error and coefficients (slopes) are expressed in px grey values.

^‡^Confidence interval for coefficients is set at 99%.

^§^Statistical significance is set at α = 1 × 10^–8^ (*P* < 1 × 10^–8^).

## Discussion

In this paper we described a novel approach for quantification of colocalization of IF signals of multiple markers on high-resolution panoramic images. For the presentation purposes, human gingiva samples were stained with several primary antibodies against specific extracellular matrix (ECM) components and common leukocyte marker CD45. Attributes of IF signal, such as the expression domain (i.e., proportion of the whole-section or tissue compartment areas covered by IF signal) and spatial gradients (variation of IF signal intensity, or mean px GV, in entire histological section), were quantified by histograms and 2D plot profiles of panoramic IF images, respectively. The data output from histograms and 2D plot profiles is suitable for parametric statistical tests even if a small number of samples is analyzed. ANOVA was used for analysis of expression domains to disclose if the overall expression of investigated factors significantly changes when compared between groups of samples. Simple and multiple linear regression were used for the analysis of spatial gradients with assumption of true linear relationship between the two or multiple investigated factors if their expressions perfectly overlap in space. Additionally, regression models were created to predict how the changes of expression of a set of investigated factors (as independent variables) affect the overall expression of the outcome marker (in this case CD45). For this type of analysis, a computational colocalization of multiple factors needs to be done where a sample 2D plot profile is used as a template (Fig. 4).

This approach to quantification of IF signals is entirely based on simple algorithms for px count and measurement of px luminance (px GV). It can also serve as an alternative to cell counting tools even though significant improvements of these tools have been recently introduced (including the new „Mastodon“ pluggin for ImageJ)^14^. The rationale behind this statement is that when IF signals (or IHC staining) get recorded on a digital image, they assume digital properties and can be perceived as a number of pixels with specific GV and location. As demonstrated here, the cellular content of histological sections can be calculated in the form of the expression domain of IF signals of DAPI nuclei marker.

**Figure 5:**
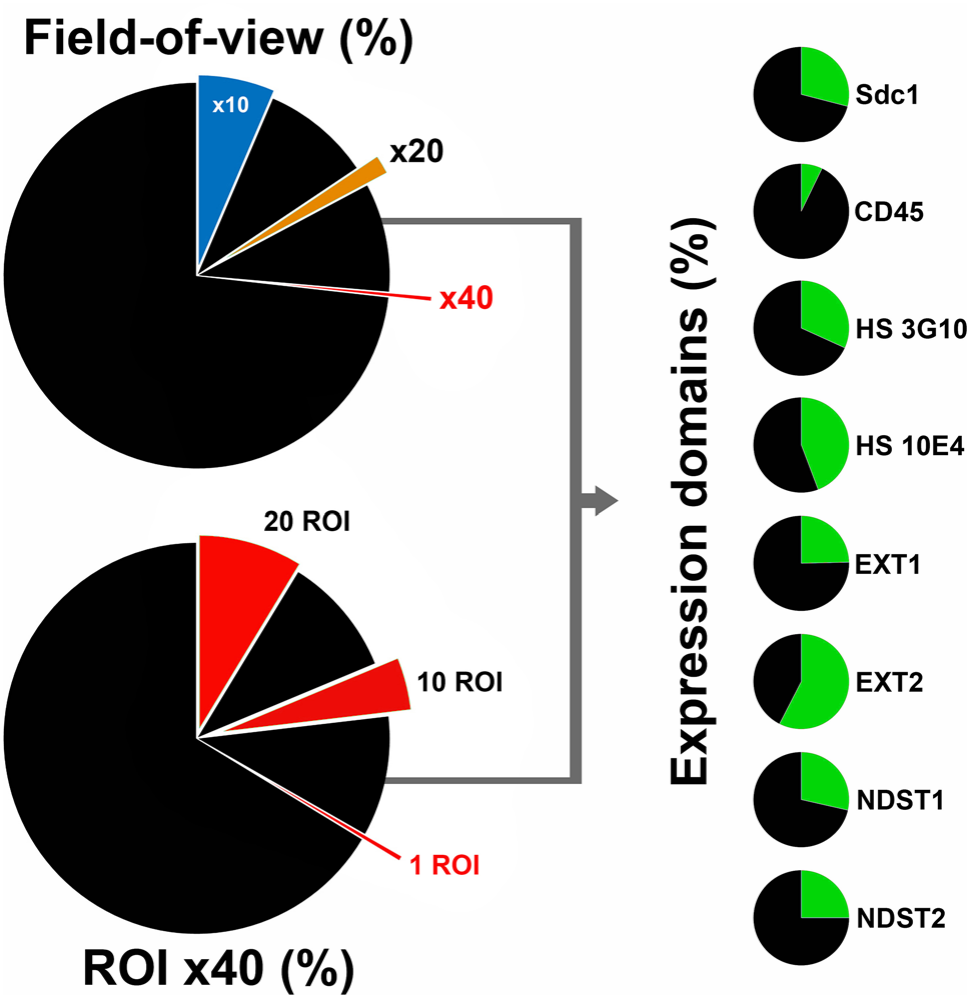
Pie-chart comparison of expression domains of investigated factors with field-of-view (magnifications × 10, × 20 and × 40) and ROIs (× 40) expressed as fraction areas (%) from the whole-section area of samples of human gingiva. Whole-section areas absent of IF signals outside of field-of-view or excluded from ROIs are presented in black color. HSPGs, enzymes for biosynthesis of HS GAGs and HS GAGs (as much as the other ECM components) are ubiquitously expressed in various tissues and have large expression domains. Thus, the traditional approach for analysis and quantification of IHC staining or IF signals in which several ROIs are selected under high magnification (× 40) might indroduce bias by excluding from the analysis significant portions of the tissue. The expression domains are presented as cumulative values which does not account for the actual spatial distribution of IF signals. Another issue is related to the size of the analyzed samples. Histological sections of human gingiva analyzed here are relatively small (between 9 mm^2^ and 12 mm^2^)—a single ROI at magnification × 40, covers only 0.44% of the whole-section area, meaning that selection of 10 ROIs (4.4%; 95.6% of whole-section area excluded from analysis) or 20 ROIs (8.8%; 91.2% of whole-section area excluded from analysis) cannot cover the entire expression domain of investigated factors. In bigger tissue samples, as the whole-section area increases, the fraction areas the from field-of-view and selected ROIs proportionally decrease. (Image created in Adobe Photoshop CC 2014, ver. 6.3; https://www.adobe.com/products/photoshop.html).

In this approach, there is no need for selection of multiple ROIs because panoramic images are analyzed entirely. The selection of ROIs prior to actual quantification of IF signals of IHC staining is the usual procedure in many semi-quantitiative and quantitative scoring systems^15–19^. While this may be sufficient when analyzing particular structures or expression of highly specific markers, it may not be adequate for quantification of ubiquitously expressed markers whose expression domains cannot be covered by several ROIs on high magnification (Fig. 5). However, the downside of IF signal quantification from the entire panoramic image is that significant effort must be invested in histo-morphometric profiling of histological sections (Fig. 1). This is mostly related to variations in tissue structure and (to some degree) to the choice of markers whose IF signals need to be analyzed. Either way, the histo-moprhometric parameters need to be quantified in detail, as these may provide a source of confounders for subsequent statistical analysis of IF. Another confounder is related to the thresholding of IF signals or separating IF signals from non-specific staining (auto-fluorescence). Therein reside the advantages of IF compared to chromogens in IHC—fluorochromes provide signals with higher dynamic range which is of utmost importance for determination of IF signal threshold. Equalization of images, i.e. the increase of dynamic range, is recognized as an important step in digital image post-processing for the purpose of signal thresholding in IF and IHC^20^. In this approach, the equalization of panoramic IF images is done simultaneously with image acquisition and not in the post-processing. Apart from the improvment of the workflow, this generally reduces the exposure of sections to excitation light, which in turn reduces the possibility of photo-bleaching of IF signals. While this is not an issue when only a few IF images need to be captured, in acquisition of panoramic IF images this is useful since the exposure of histological sections to excitation light is prolonged..

In quantification and analysis of spatial gradients of IF signals, the influence of the very shape of histological sections must not be neglected. Since the quantification of IF signals is done on serial histological sections, a certain margin of sampling error might be introduced because no two sections from the same sample are completely identical. The simplest way to check if the difference in section shape within a sample can confound the subsequent statistical analysis of spatial gradients is by correlating 2D plot profiles of DAPI IF signals from consecutive histological sections. From tissue samples analyzed here, up to 30 consecutive sections with DAPI IF signals display almost perfect shape ad cell content correlation in single and multiple linear regression models (Table 1). This provides us with the estimate of the total number of different factors whose IF signals' spatial gradients can be quantified, co-localized and correlated using 2D plot profiling if successfully stained on consectuive sections. It should also be noted that when using 2D plot profiles for quantification of spatial gradients of IF signals, the formatting and precise alignment of panoramic IF images is of paramount importance (Figs. 3, 4). In this approach, image alignment was done semi-automatically by action presets customized exclusively for each sample in Adobe Photoshop CC 2014 ver. 6.3 (Adobe, San Jose, CA, USA), although there is a variety of pattern recognition software currently available that might be used to improve the workflow^21^.

In the past the technology was developed as a way to simultaneously co-localize and identify unique combinations of multiple markers on a cellular or tissue level—multi-epitope ligand cartography (MELC) is based on direct IF immunolabeling and consecutive imaging of tens to hundred of proteins in the same field-of-view^22,23^. In MELC, the IF immunolabeling of multiple markes is performed on the same histological section by repeating cycles of IF staining. Image acquisition and photo-bleaching of captured IF signals in situ are performed before the immunolabeling of another marker takes place. Along with the unified coding system in which the IF signals from multiple markes are detected as spatial signal maps and presented as binary vectors, the main advantage of MELC is that by using a relatively small number of samples it can identify hierarchical organization of complex protein networks with great precision. Additionally, by application of MELC, even the histomorphometric parameters of tissues can be analyzed as is nicely demonstrated in studies on effects of cancer heterogeneity on effectiveness of various cancer therapeutics^24^. On the downside, the protocol for MELC is fully automated and requires the use of sophisticated equipment which may be out-of-reach for small laboratories in academia settings.

The spatial distribution of proteins and other classes of molecules is important determinant of their biological function in tissues. In interpretation of data from IHC-based methods every effort is made to connect those two. With no disregard to well-known limitations of IHC, the validity of such interpretation is significantly related to the ability of researchers who use IHC to precisely quantify expression of investigated markers^5,25^. Another issue is the traditionally small amount of markers that can be simultaneously co-localized and visualized and there is every need to go beyond the confinements of double immuno-labeling. The approach presented here is not the first one to improve that, but it does attempt to do so based on the standard staining protocols. It also aims to improve on the co-occurrence/correlation bias which is recognized in standard px-based approaches to colocalization of IF and counteracted by application of various tests such as Costes' randomization test^33–35^. Namely, the co-occurrence/correlation bias, as recognized by the standard px-based approaches to colocalization of IF which utilize histograms, is effectively eliminated when using 2D plot profiles for colocalization of IF from multiple markers as described here. 2D plot profiles contain more information than histograms, and when integrated in regression matrices, correlation analysis reflects both the overlap and variation of intensity of IF signals in space. Thus, more robust statistical models on functional importance of colocalization of multiple markers can be created. The number of factors that can be included in these models is sufficient to completely or partially reconstruct the signaling pathways or elements of regulatory networks in silico using data from ontologic bases as the template. Then, the ultimate test of the validity of this approach to quantification of IF can be conducted—by seeing if the predictions from statistical models can be replicated in actual live experiments.

## Methods

For this study we used samples of human gingiva from the archival collection of histological slides at the Department of Anatomy, Histology and Embryology (School of Medicine, University of Split). Gingiva samples were obtained from patients diagnosed with general destructive periodontitis. Patient screening and recruitment were done in accordance with the guidelines for classification of periodontal and peri-implant diseases as described previously^26,27^. The sampled tissue was free gingiva which is a part of masticatory gingiva that spans from the gingival margin to the ridge of the alveolar bone. Procurement and processing of tissue samples were approved by the Ethical and Drug Committee of School of Medicine, University of Split (Class: 003-08/17-03/0001, No: 2181-198-03-04-17-0043) in accordance with Helsinki Declaration^28^. To facilitate the proper tissue orientation during the paraffin embedding, vestibular (labial/buccal) aspects of gingiva samples were marked by waterproof color immediately prior to sampling. Fixation in 4% paraformaldehyde took between 24 to 48 h. The samples were then cut in serial 5 µm thick sections and mounted on glass slides (three sections per slide). Every 10th slide was stained with hematoxylin/eosin (H/E) to confirm the presence and preservation of the structures of interest (gingival epithelium, gingival sulcus epithelium and subepithelial stroma).

### Immunofluorescence (IF) staining

Deparaffinization and IF staining were performed following the standard protocol in our laboratory^29,30^. Background blocking was done using Abcam Protein Block (ab64226; Abcam, UK) for 25 min after the antigen retrieval. The primary antibodies used for IF staining were: mouse-monoclocal anti-Sdc1 (syndecan 1) (1:100; ab34164; Abcam, UK), rabbit polyclonal anti-EXT1 (exostosin 1) (1:100; ab126305; Abcam, UK), rabbit polyclonal anti-EXT2 (exostosin 2) (1:50; ab102843; Abcam, UK), rabbit polyclonal anti-NDST1 (bifunctional heparan sulfate N-deacetylase/N-sulfotransferase (1) (1:50; ab129248; Abcam, UK), rabbit polyclonal anti-NDST2 (bifunctional heparan sulfate N-deacetylase/N-sulfotransferase (2) (1:100; ab151141; Abcam, UK), mouse monoclonal anti CD45 (common leukocyte antigen/inflammatory cell marker) (1:200; ab8216; Abcam, UK), mouse monoclonal anti-HS 3G10 (heparan sulfate glycosaminoglycan, 3G10 epitope) (1:100; 370260-1; Seikagaku Corp., Japan) and mouse monoclonal anti-HS 10E4 (heparan sulfate glycosaminoglycan, 10E4 epitope) (1:100; 370255-1; Seikagaku Corp., Japan). Sections were incubated with primary antibodies for 24 h at 4 °C. For application of anti-HS 3G10, sections had to be pre-treated with Heparinase III enzyme (0.02 IU/50 µl) (Seikagaku Corp, Japan) at 37 °C for 2 h. Secondary antibodies were used at dilution 1:400: anti-mouse Alexa Fluor 488 (GREEN; ab150105; Abcam, UK) and anti-rabbit Alexa Fluor 488 (GREEN; ab150077; Abcam, UK). Cell nuclei were stained with diamidino-2-phenylindole (DAPI). Staining of samples of oral mucosa from maxillary tuberosity was performed as positive control. Expression patterns (nuclear/non nuclear) of IF signals were examined by the intensity correlation analysis (ImageJ Coloc Finder pluggin) as previously described^31,32^. IF signals of all primary antibodies displayed non-nuclear expression patterns (data not shown).

### Image acquisition and processing—H/E panoramic images

Orientation slides stained with H/E were examined under Olympus BX40 light microscope equipped with standard digital camera (Olympus DP27, Olympus, Tokyo Japan), and area-scan high-resolution digital camera (Basler aceA2500-14gm, Basler, Germany) for manual slide scanner (Microvisioneer, Esslingen am Necker, Germany). H/E panoramic images were captured at magnification ×20 (exposure time: 8 ms; ISO: 100), exported as JPEG files and post-processed in Adobe Photoshop CC 2014 ver 6.3 (Adobe, San Jose, CA, USA). Orientational alignment was done by the action presets customized for every sample individually because sections from different samples vary in size and shape. The presets contain 4 basic operations for orientational alignment („Transform“, „Rotate“, „Crop“ and „Merge“) and enable execution of these operations by a single click. The overall size of H/E panoramic images (600 MB on average) was reduced by converting them from TIFF to JPEG format on a high-resolution background (600 dpi) in order to minimalize image detail loss and to preserve data storage space since these images were only used for preliminary histomorphometry. Epithelial tissue compartment (gingival epithelium, gingival sulcus epithelium) and stromal tissue compartment were masked using graphic pen tablet (Wacom Intuos PRO, Wacom Co., Saitama, Japan). Whole-section area and areas of tissue compartments were measured by Magic Wand Tool/Histogram to be expressed as absolute values in px (number of px) (whole-section area), and as relative values (proportion of the whole-section area) expressed in percentages (%) (areas of tissue compartments). These measurements were subsequently used to calculate the group averages of whole-section areas and tissue compartment (epithelial, stromal) fraction areas. The same procedure was performed on panoramic IF images.

### Image acquisition—panoramic IF images

**Figure 6:**
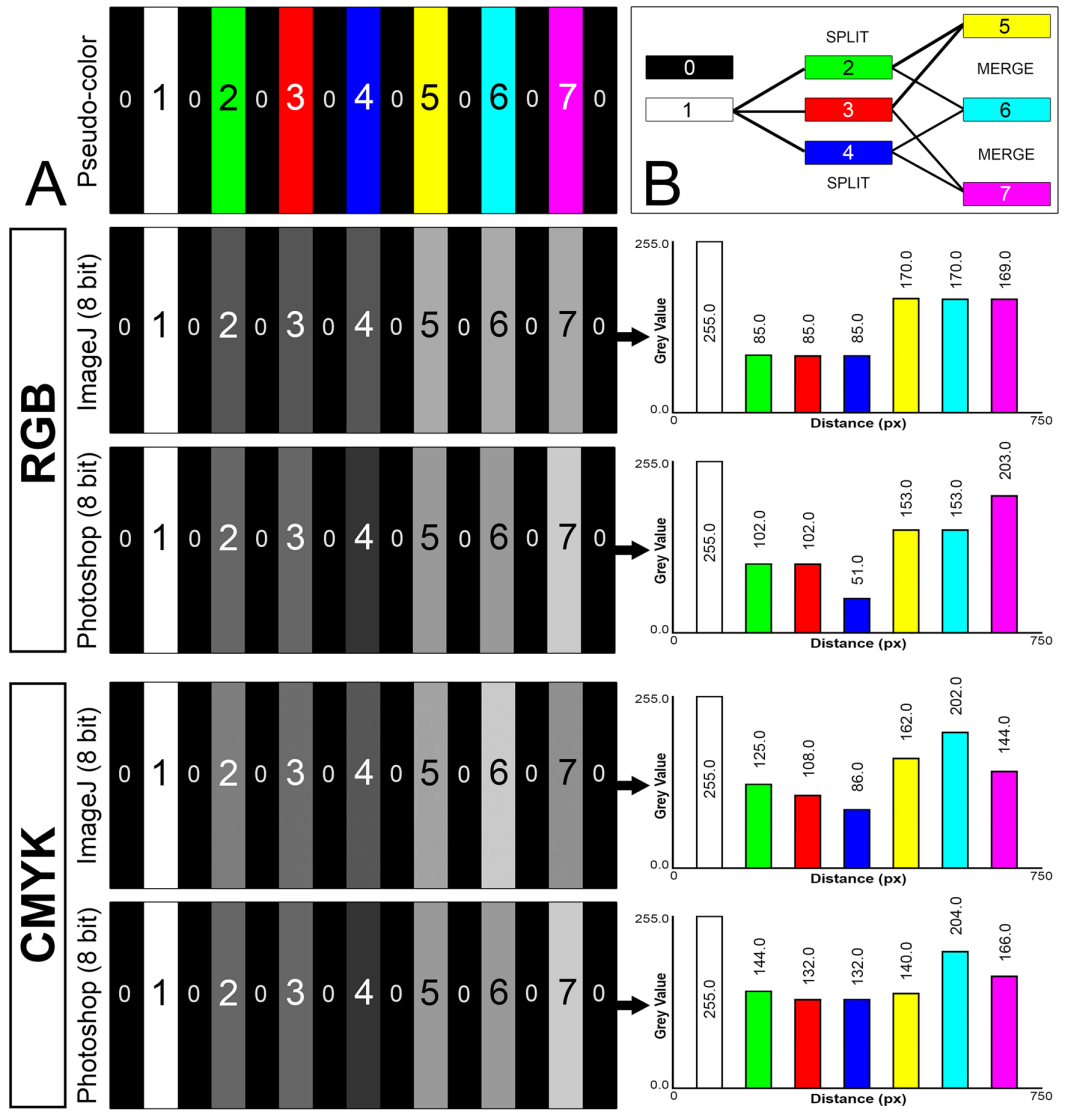
750 × 150 pixel (px) color step-tablet (**A**) comprised of seven panels separated by black spaces (0). White panel (1) is pseudo-colorized in green (2), red (3) and blue (4) with three different merge combinations: yellow (green/red merge) (5), cyan (green/blue merge) (6) and magenta (red/blue merge) (7) as shown on scheme (**B**). Thick arrows point to 2D plot profiles of average px grey values (GVs) measured in left–right direction for each desaturated (converted to black & white) step-tablet. The average px GV is calculated from a 0 to 255 scale of all pxs in a 1 px-wide columns. Each panel from step-tablets contains the total number of 50 1 px-wide columns, and each column contains 150 pxs. Thus, for white panel (1), the average px GV is consistently 255.0 since all pxs belonging to white panel have GV of 255. Conversely, the average GV of black spaces is 0.0 since all black pxs have GV of 0. It should be noted that the average grey values for all pseudo-colorized panels are lower than the GV of the white panel. That is because the image analysis software discriminates colors in different shades of grey and prior to the analysis of colorized (or pseudo-colorized) image, desaturation (i.e. conversion to 8-bit black & white image) must be performed. The output average GV measurements of pseudo-colorized panels can also be affected by the inherent differences of algorithms for color desaturation applied in different software (see step-tablets for comparison between different software) and by different color modes such as Red–Green–Blue (RGB) and Cyan–Magenta–Yellow–Key (CMYK). These points should be considered for quantification IF signals based on measurements made on color images, especially with regard to double IF. (Image created in Adobe Photoshop CC 2014, ver. 6.3; https://www.adobe.com/products/photoshop.html).

IF stained slides were examined under Zeiss Axio Observer inverted epifluorescence microscope (Carl Zeiss Microscopy GmbH; Jena, Germany) equipped with Zeiss Axiocam 506 digital color camera (Carl Zeiss Microscopy GmbH, Jena, Germany) set for full frame resolution (2752 × 2208 px) which enables the acquisition of photomicrographs in original black & white (8-bit depth, TIFF), as well as in color format (Pseudo-colorizer module, sRGB color mode). Acquisition and stitching of photomicrographs into panoramic IF images were done simultaneously using Multi-channel and Panorama modules in ZEN 2.5 software (Carl Zeiss Microscopy GmbH; Jena, Germany), respectively. The settings for image histogram equalization (background subtraction and contrast adjustment) were saved as the main calibration preset executed automatically during the entire procedure of photomicrograph acquisition. Therefore, the panoramic IF images were rendered to display full dynamic range of px GV on a scale from 0 (pure black) to 255 (pure white). Depending on the fluorescence channel, this also allowed us to make 2- to 5-fold reduction of the overall exposure time for photo-micrograph acquisition and consequently avoid the risk of photo-bleaching of IF signals. The exposure time in Multi-channel module for blue channel (DAPI IF signals) was set at 35 ms, whereas for the green channel (IF signals of primary antibodies) was set at 800 ms. For the stitching of photomicrographs (tiles) into panoramic IF images, tiles were captured sequentially by manual navigation along x/y axes guided by the on-screen live view of DAPI channel with minimal 20% overlap (x-axis) and 10% maximal shift (y-axis) between individual tiles. Panoramic IF images were closely inspected for quality (tiles alignment, stitching artefacts) and stored in raw format as CZI files (Zeiss proprietary file format) that contains original data and metadata. CZI files of each panoramic IF image were then exported in multiple TIFF format files—original black & white 8-bit, pseudo-colorized 8-bit and merged 16-bit). Only the original black & white 8-bit panoramic IF images were post-processed and used for further analysis. Namely, the pseudo-colorization reduces the overall dynamic range of IF signals making the adjustment of cut-off signal threshold (i.e. discrimination between the true IF signals and autofluorescence) and consequently the quantification of IF signals less precise. The reduction of dynamic range occurs because every software for image editing and/or image analysis „perceives“ colors as different shades of grey and by default converts color images to black & white images. How application of different software and even different image modes can affect the measurement of signal intensity (as output of px GV) is shown on a color step-tablet (Fig. 6). In order to confirm that IF signals display full dynamic range, four-color heatmaps of panoramic IF images were made, where each color represents a range of px GV as follows: BLUE (10–49; weak intensity), GREEN (50–149; moderate intensity), RED (150–254; strong intensity), YELLOW (255; very strong intensity). The protocol for the making of heatmaps was previously described^31^.

### Post-processing of panoramic IF images

**Figure 7:**
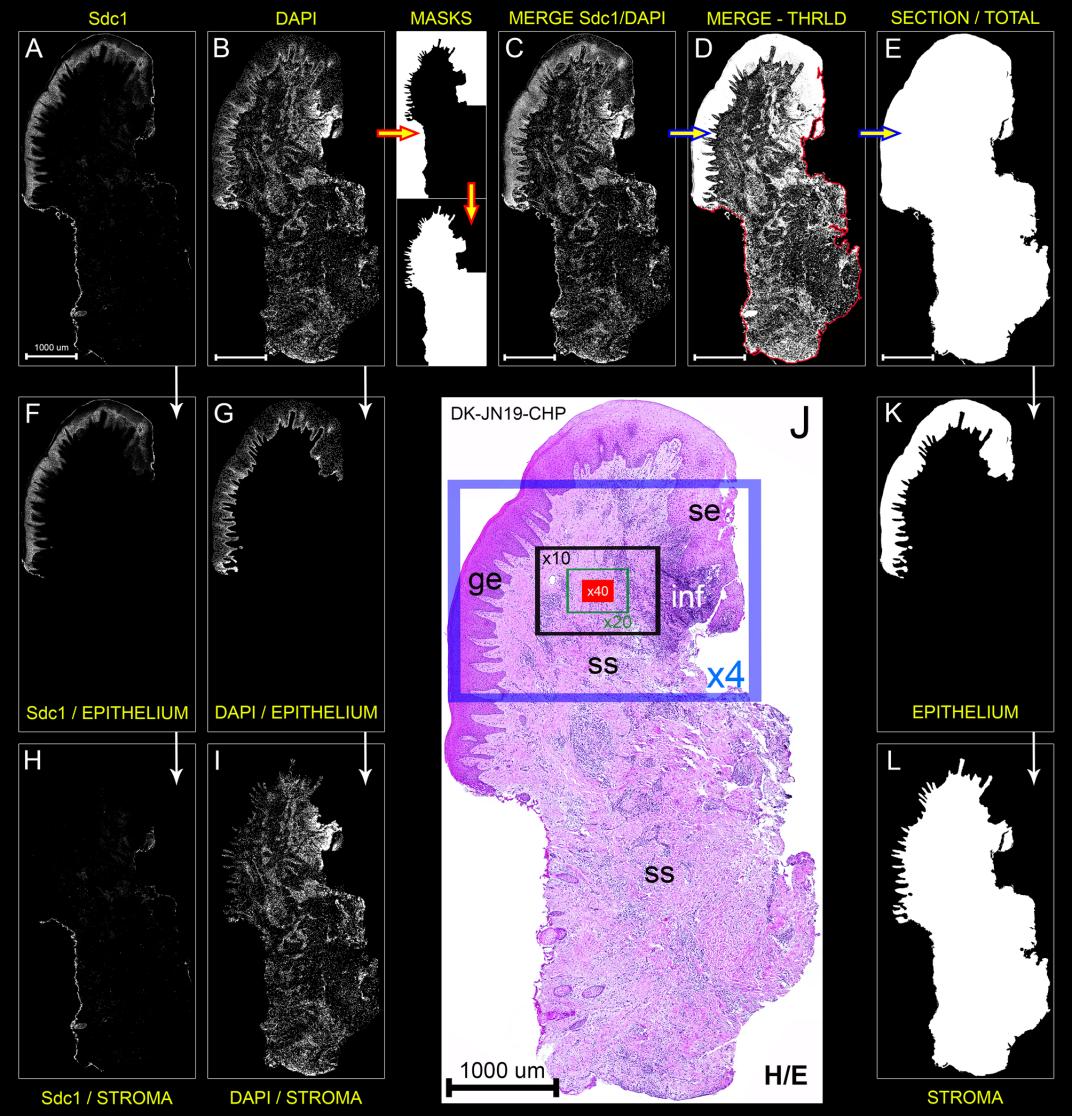
Processing protocol for 8-bit (black & white) high-resolution panoramic IF images (dimensions: 6250 × 12,000 px) of histological section of gingiva from patient with advanced generalized periodontitis (sample: DK-JN19-CHP) containing anti-Sdc1 IF staining (**A**) and DAPI nuclear staining (**B**). Designations: *ge* gingival epithelium, *se* gingival sulcus epithelium, *ss* subepithelial stroma, *inf* inflammatory infiltrate, *H/E* hematoxylin/eosin staining. (*Magnification:* × 10 (**A**–**I**,**K**,**L**) and × 20 (**J**); *scale bar:* 1000 µm). By tracing DAPI image as a template (background image), blending masks are made (thick yellow/red arrows) which will be applied for separation of epithelial compartment (includes both ge and se) and stromal compartment (thin white arrows) on Sdc1, DAPI and whole section area panoramic IF images. Merging is done in Adobe Photoshop CC 2014 ver 6.3 (Adobe, San Jose, CA, USA) using „DARKEN “ blending which enables visibility only of those parts of background under the white area of blending mask (thin white arrows; **F**–**I**,**K**–**L**). To produce a whole-section area panoramic image (**E**), Sdc1 and DAPI panoramic IF images are merged (**C**), and then set to the lowest possible threshold (1 px GV) (**D**) in order to partially fill in-section areas which contain no staining. The remaining gaps on the outer edges of newly produced whole-section area panoramic image (around **ss** where the oveall texture of tissue is loose) are closed wih „BRUSH “ tool (**D**, red color). This enables the precise selection of the total section area using „MAGIC WAND “ tool and subsequent filling of the whole-section area with pure white by „FILL “ option (**E**, thick yellow/blue arrows). Thus, the panoramic IF images are processed for quantification of histomorphometric parameters (surface size, celularity, tissue compartment areas) and IF signals of investiagted factors (expression domain, spatial gradient) in the whole-section scale or in particular tissue compartments. In contrast to standard procedures for quantification of IHC and IF signals, there is no need for selection of multiple ROIs on high magnification (× 40). (**J**; field-of-view at magnifications × 4 (blue frame), × 10 (black frame), × 20 (green frame), × 40 (red frame)). (Image created in Adobe Photoshop CC 2014, ver. 6.3; https://www.adobe.com/products/photoshop.html).

Post-processing of panoramic IF images was performed entirely in Adobe Photoshop CC ver. 6.3 (Adobe, San Jose, CA, USA). Orientational alignment of panoramic IF images was done in a similar way described for panoramic H/E images. Since the panoramic IF images were taken at ×10 magnification at full-frame resolution (and thus were comprised of less individual photo-micrographs), there was no need for significant size reduction compared with panoramic H/E images. However, some formatting was still needed since ImageJ cannot load images which exceed certain dimensions limit (~120 000 000 px^2^) irrespective of the file format. For the creation of whole-section area panoramic images, IF signal and corresponding DAPI panoramic IF images were merged („Lighten“ blending mode) and then thresholded at the lowest px GV of 1 rendering them as binary images with only two px GV values (0 and 255). The remaining black gaps at the interface between the background and tissue section on the newly produced merged images were traced with „Brush“ tool in order to enable the selection of the whole section by „Magic Wand“ tool. Then, „Total fill“ option was applied to fill the whole section area with pure white color. Blending masks for tissue compartments (epithelial/stromal) were made by tracing the interface between the epithelial and stromal tissue with „Brush“ tool. In this case, DAPI panoramic images were used as templates. IF signal panoramic images and whole-section area panoramic images were each blended with masks in „Darken“ blending mode. „Darken“ blending mode compares the px GV (luminance) of each pixel from the background and foreground blending layer and renders visible either the background or foreground blending layer px dependening on which one is darker. In case the two px are of equal GV, the background px remains visible after blending. Mathematical function for „Darken“ blending mode is: *R* = min{*F,B*} (*R* – resultant pixel; *F*—blending foreground pixel; *B* = background pixel). For each pair of IF signal and DAPI panoramic images, nine additional panoramic images were created—epithelial and stromal IF signal, epithelial and stromal DAPI and binary 8-bit images of the whole section area, two blending masks, epithelial and stromal compartment area (Fig. 7).

### IF signal thresholding, histograms and 2D plot profiling

For the IF signal thresholding, we first made the measurement of the total background px GV at low GV thresholds (1–10) using „Threshold“ option in Adobe Photoshop CC 2014 ver 6.3 (Adobe, San Jose, CA, USA). After that, a ROI on panoramic image which contains weak IF signals is selected (Fig. 2C) and inspected for a range of px GV (10–50) (Fig. 2O), cropped and further processed in at least five 8-bit threshold images at specific threshold values (for example at 10, 20, 30, 40 and 50). 2D plot profiles of the original ROI and threshold ROIs were made in ImageJ and used for correlation analysis—the IF signal threshold cut-off value equals to the threshold value of the threshold ROI which best correlates with the original ROI. Because we used 2D plots of threshold binary images containing px with GV of either 0 or 255, the correlation is strictly proportional to the amount of spatial overlap (or location) of the patterns on threshold images (Fig. 2D,G,J,M,P) with IF signals on the original ROI (Fig. 2C; Supplementary material: Dataset 3). Once the histograms of panoramic IF signal images are created („Histogram“ option in ImageJ), px counts for GVs below the threshold cut-off can be discarded (Fig. 2). The histograms have the uniform output of 256 values irrespective of the type or size of the image. This enables the presentation of expression domains of IF signals in the form of fractional px counts for each px GV. It also makes more sense because expression domain is generally comprised of signals with a range of intensities—if the expression domain is simply presented as a single cumulative value, a lot of information is lost. Once the IF signal thresholding was complete, the original panoramic IF images were further edited in order to „physically“ exclude px with GV below the threshold cut-off. Each original panoramic IF signal images was merged with counterpart inverted threshold cut-off panoramic IF image using „Darken“ blending mode. As such, the original panoramic IF signal images are fully processed for the analysis of spatial gradients of IF signals. Again, the spreadsheet table of top-down (vertical) 2D plot profile of the panoramic IF image is extracted in ImageJ and calibrated to fit the µm scale (at ×10 magnification 1 px = 0.53937 µm) (Fig. 3A–D). 2D plot profiles basically list the average px GVs per 1 px-wide rows. In contrast to histograms, this means that the total number of output values depends on image dimensions. For the panoramic IF images of gingival samples taken at magnification ×10, the average 2D plot profile output is between 8000–12000 values. 2D plot profiles are then used for *in silico* colocalization of multiple IF signals and correlation analysis of their spatial gradients.

### Statistical analysis

The attributes of IF signals were fully quantified and presented as continuous variables. Therefore, only parametric tests were used for the statistical analysis. Histomorphometric parameters of gingiva samples (whole section area, fraction areas of tissue compartments, cellularity) were analyzed by descriptive statistics and *t-*test. The level of significance for basic histomorphometric profiling of samples was set at α = 0.1 (*P* < 0.1) since these parameters were mostly presented as cumulative (single) values. For the analysis of expression domains of IF signals, single factor ANOVA was used with the level of significance set at α = 0.01 (*P* < 0.01). Correlation of spatial gradients of IF signals was analyzed by simple and multiple linear regression with the level of significance set at α = 1×10^−8^ (*P* < 1×10^−8^). Based on multiple linear regression, a model was created in order to assess what kind of effect might each investigated factor (HS GAG, EXTs, NDSTs) exert on the presence of inflammatory infiltrate (CD45 set as main dependent variable) by means of VKO, i.e. replacing all values in top-down 2D plots (T-D plots) of individual factors with 0. For T-D plots image is scanned in direction from the top to the bootom (along image height). The reason for choosing linear regression model was following: when the values from T-D plots of IF signals from two different factors are plotted on the x/y axis graph in the same sequence as they appear on the T-D plot, but in a way that T-D plot values from factor 1 go on x-axis (independent variable), and those of factor 2 on y-axis (dependent variable), they would conform to a linear function if they perfectly co-localize in space. Conversely, the more scatter appears on the x/y axis graph, the less two factors co-localize in space. The refinement of multiple linear regression model was done by non-linear polynomial regression (6th order). Statistical tests and calculations were performed in Microsoft Office Excel 2016 (Microsoft Corp., Redmond, WA, USA). Datasets used for statistical analysis are publicly available (Supplementary material).

### Ethical approval

Procurement and processing of tissue samples used in this study were approved by the Ethical and Drug Committee of School of Medicine, University of Split (Class: Class: 003-08/17-03/0001, No: 2181-198-03-04-17-0043).

## Supplementary Information

Supplementary Information 1.

Supplementary Information 2.

Supplementary Information 3.

Supplementary Information 4.

Supplementary Information 5.

Supplementary Information 6.

Supplementary Information 7.

## References

[1] Huss R, Coupland SE (2020). Software-assisted decision support in digital histopathology. J. Pathol..

[2] Tummers M, Thesleff I (2009). The importance of signal pathway modulation in all aspects of tooth development. J. Exp. Zool. Part B Mol. Dev. Evol..

[3] Taylor CR, Levenson RM (2006). Quantification of immunohistochemistry—issues concerning methods, utility and semiquantitative assessment II. Histopathology.

[4] Riber-Hansen R, Vainer B, Steiniche T (2012). Digital image analysis: A review of reproducibility, stability and basic requirements for optimal results. APMIS Acta Pathol. Microbiol. Immunol. Scand..

[5] Roper JA, Williamson RC, Bass MD (2012). Syndecan and integrin interactomes: Large complexes in small spaces. Curr. Opin. Struct. Biol..

[6] Rozario T, DeSimone DW (2010). The extracellular matrix in development and morphogenesis: A dynamic view. Dev. Biol..

[7] Bernfield M (1992). Biology of the syndecans: A family of transmembrane heparan sulfate proteoglycans. Annu. Rev. Cell Biol..

[8] Ćavar I, Kero D (2020). Correlation of the expression of hyaluronan and CD44 with the presence of gingival inflammatory infiltrate in advanced generalized periodontitis. ST-OPEN.

[9] Barritault D (2017). RGTA((R)) or ReGeneraTing agents mimic heparan sulfate in regenerative medicine: from concept to curing patients. Glycoconj. J..

[10] Esko JD, Selleck SB (2002). Order out of chaos: Assembly of ligand binding sites in heparan sulfate. Annu. Rev. Biochem..

[11] Presto J (2008). Heparan sulfate biosynthesis enzymes EXT1 and EXT2 affect NDST1 expression and heparan sulfate sulfation. Proc. Natl. Acad. Sci. U.S.A..

[12] Talsma DT (2018). Endothelial heparan sulfate deficiency reduces inflammation and fibrosis in murine diabetic nephropathy. Lab. Investig..

[13] Zhang X, Wang F, Sheng J (2016). "Coding" and "Decoding": Hypothesis for the regulatory mechanism involved in heparan sulfate biosynthesis. Carbohyd. Res..

[14] Xie Y (2015). Deep voting: A robust approach toward nucleus localization in microscopy images. Med. Image Comput. Comput. Assist. Interv..

[15] Boric K (2019). Expression of apoptotic and proliferation factors in gastric mucosa of patients with systemic sclerosis correlates with form of the disease. Sci. Rep..

[16] Kero D (2014). Expression of cytokeratin 8, vimentin, syndecan-1 and Ki-67 during human tooth development. J. Mol. Histol..

[17] Kotsovilis S (2010). Syndecan-1 immunohistochemical expression in gingival tissues of chronic periodontitis patients correlated with various putative factors. J. Periodontal Res..

[18] Matos LL (2006). Immunohistochemistry quantification by a digital computer-assisted method compared to semiquantitative analysis. Clinics.

[19] Sanchez-Romero C, Bologna-Molina R, Mosqueda-Taylor A, Paes de Almeida O (2016). Immunohistochemical expression of GLUT-1 and HIF-1alpha in tooth germ, ameloblastoma, and ameloblastic carcinoma. Int. J. Surg. Pathol..

[20] Waisberg J (2016). Immunohistochemical expression of heparanase isoforms and syndecan-1 proteins in colorectal adenomas. Eur. J. Histochem. EJH.

[21] Parfitt GJ (2012). A novel immunofluorescent computed tomography (ICT) method to localise and quantify multiple antigens in large tissue volumes at high resolution. PLoS ONE.

[22] Schubert W (2003). Topological proteomics, toponomics, MELK-technology. Adv. Biochem. Eng. Biotechnol..

[23] Schubert W (2006). Analyzing proteome topology and function by automated multidimensional fluorescence microscopy. Nat. Biotechnol..

[24] Park CC (2013). Rapid and automated multidimensional fluorescence microscopy profiling of 3D human breast cultures. Integr. Biol. Quant. Biosci. Nano Macro.

[25] Kero D, Saraga-Babic M (2016). Odontogenesis—a masterful orchestration of functional redundancy or what makes tooth bioengineering an intrinsically difficult concept. J. Stem Cell Res. Therap..

[26] Duplancic R (2019). Syndecans and enzymes for heparan sulfate biosynthesis and modification differentially correlate with presence of inflammatory infiltrate in periodontitis. Front. Physiol..

[27] Papapanou PN (2018). Periodontitis: Consensus report of workgroup 2 of the 2017 world workshop on the classification of periodontal and peri-implant diseases and conditions. J. Clin. Periodontol..

[28] Williams JR (2008). The declaration of Helsinki and public health. Bull. World Health Organ..

[29] Kero D (2016). Involvement of IGF-2, IGF-1R, IGF-2R and PTEN in development of human tooth germ—an immunohistochemical study. Organogenesis.

[30] Kero D (2015). Analysis of expression patterns of IGF-1, caspase-3 and HSP-70 in developing human tooth germs. Arch. Oral Biol..

[31] Kero D, Bilandzija TS, Arapovic LL, Vukojevic K, Saraga-Babic M (2018). Syndecans and enzymes involved in heparan sulfate biosynthesis and degradation are differentially expressed during human odontogenesis. Front. Physiol..

[32] Kero D (2017). Regulation of proliferation in developing human tooth germs by MSX homeodomain proteins and cyclin-dependent kinase inhibitor p19(INK4d). Organogenesis.

[33] Costes SV (2004). Automatic and quantitative measurement of protein-protein colocalization in live cells. Biophys. J..

[34] Aaron JS, Taylor AB, Chew TL (2018). Image co-localization—co-occurrence versus correlation. J. Cell Sci..

[35] Dunn KW, Kamocka MM, McDonald JH (2011). A practical guide to evaluating colocalization in biological microscopy. Am. J. Physiol. Cell Physiol..

